
JOURNAL INFORMATION
==============================
NLM Title Abbreviation: ScientificWorldJournal
Journal ID: ScientificWorldJournal
NLM Journal ID: 101131163
Journal ID: TSWJ

The Scientific World Journal

ISSN: 2356-6140
EISSN: 1537-744X
Publisher: Wiley

ARTICLE INFORMATION
==============================
PMCID: PMC5901359
PMID: 29861698
DOI: 10.1100/tsw.2007.263
Article ID: 850239
Article version: 1
Subjects: Introductoin

Current Challenges in Spina Bifida Care

Joseph David B.
Division of Urology, University of Alabama, Birmingham, AL, USA
Publication date: 2007
Electronic publication date: 2007 Dec 10
Volume: 7
First page: 1930
Last page: 1931
Copyright: Copyright © 2007 David B. Joseph.
Copyright year: 2007
License: This is an open access article distributed under the Creative Commons Attribution License, which permits unrestricted use, distribution, and reproduction in any medium, provided the original work is properly cited.
License URL: https://creativecommons.org/licenses/by/3.0/

==============================
Introduction
Special Issue: Challenges in Spina Bifida
*TheScientificWorldJOURNAL*, (2007) **7**, 1930–1931
TSW Urology
ISSN 1537-744X; DOI 10.1100/tsw.2007.263

TheScientificWorldJOURNAL
www.thescientificworld.com

### Current Challenges in Spina Bifida Care

David B. Joseph
*Division of Urology, University of Alabama, Birmingham, AL, USA*

**E-mail:** david.joseph@ccc.uab.edu

*Published December 10, 2007*

---

Spina Bifida is one of the most common and challenging birth defects. The secondary effects caused by the neurologic deficits cross multiple disciplines in medicine, education and social well being, increasing the complexity of the condition. While great progress has been achieved in prolonging the lifespan and improving the quality of life for individuals with Spina Bifida, it is recognized that there are new challenges ahead. A multidisciplinary conference inclusive of healthcare workers, psychologists, educators and other interested disciplines was held in 2003 to investigate the current state of care and determine if care was valid and supported by scientific evidence*. It became obvious that much of our current management has been positive, but unfortunately little if any is supported by strong scientific evidence.

Neurosurgical advancements, including back closure, improvements in shunting and treatment of the Chiari malformation, have prolonged the early childhood and teen years only to unmask the difficulties of renal compromise due to the secondary problems of the neurogenic bladder. Pediatric urology has recognized the need to rehabilitate the bladder through a multifaceted approach that includes, medical and pharmacologic management, intermittent catheterization, and often times surgical reconstruction. Now it is rare for an individual with Spina Bifida to progress to renal failure. Urologic advances and successes have helped to prolong the lives of affected individuals to adulthood. But with every success new challenges are exposed in other areas. All health care workers, in particular the urologist, must be aware of the new challenges presented in the management of individuals with Spina Bifida. This special edition of TSW Urology explores a few of the Current Challenges in Spina Bifida Care.

An overwhelming challenge recognized for the successful management of Spina Bifida is the establishment of a multidisciplinary approach to the health and well being of the individual. It is easy for one discipline to lose site of the overall management due to a focused effort on their own subspecialty. The success achieved through the organization of a multi-disciplinary clinic is due to continuity in care among specialties. Timothy J. Brei, a national leader in the organization and management of multidisciplinary clinics, has direct experience running one of the largest Spina Bifida clinics. His interest and expertise in establishing national guidelines and standards, positions him perfectly to introduce us to the current challenges of organizing and maintaining a multi-specialty clinic. Dr. Brei shares with us the importance of using a multidisciplinary approach and outlines future hurdles that must be overcome in order to improve upon our current systems.

Coordination of care is the key to a successful clinic. It is recognized in most areas of medicine that a strong partnership with the non-physician coordinator is essential. Mary Jo Dunleavy, shares her expertise as a nursing coordinator and discusses the multiple facets required of the coordinator including his/her role as a counselor, educator, triage specialist and social director. The role of the coordinator becomes personal and requires a special individual who has the conviction to run a clinic efficiently and the compassion to provide special individualized care.

©2007 with author.

Urologic management is reaching a plateau regarding reconstructive measures. The 2003 meeting on evidenced based care clearly indicated that urologic care lacks sound supporting science. Drs. Jane Lewis and Earl Cheng take a look into the future of using autologous tissue for reconstruction. In addition, they recognize our current therapy with augmentation targets the secondary changes of the neurogenic dysfunction and not the primary defect. They share with us advances in neuromodulation that may open new avenues of research and treatment.

Even with our limitations in finding supportive scientific evidence for current management, it is clear that the lifespan of individuals with Spina Bifida has been prolonged. The increased lifespan has exposed a significant deficit in even the best of multi-disciplinary clinics. That is the ability to provide adequate care to the "aging" population of adults with Spina Bifida. Transitioning care from pediatric centers to adult institutions is a complex endeavor met with apprehension from adult healthcare providers and the patients themselves. Dr. Sue Mukherjee guides us through challenges uncovered in transitioning care to an adult environment. Establishing adult care centers has taken on national attention and must prosper in order to accommodate the advancing generation.

A significant limitation of adult management is the lack of expertise in both female and male health care management. There are few adult healthcare providers who have a thorough understanding of the special needs required by adults with Spina Bifida particularly if they have undergone multiple prior urologic and non-urologic operative procedures. Female healthcare, particularly basic primary gynecologic care for disabled women is lacking. Knowledge regarding sexuality and fertility in both sexes is also limited. Drs. Jackson, and Rovner are national authorities on female and male healthcare respectively and they, along with Dr. Bong, introduces us to important challenges of adult management directed to improve an individual's health and quality of life.

There are significant financial challenges that hinder us from achieving many of our goals whether it be research to establish scientific evidence or support for organization of existing clinics and the creation of adult centers. Harnessing governmental financial support is daunting to say the least and consider by most healthcare workers to be politically beyond their reach and all too often forgotten. Cindy Brownstein, CEO of the Spina Bifida Association and Spina Bifida Foundation shares with us a primer on patient advocacy. She reviews how an organized approach can effectively become translated into governmental funding. A single individual carries a vote and that vote generates a great deal of interest from local politicians. Ms. Brownstein shows how the voice of a healthcare worker can resonate positively.

I would like to thank each author for giving of their time and expertise in order to help educate us on some of the Challenges in Spina Bifida Care. I greatly appreciate the opportunity presented by TSW Urology to bring this dialogue forward. I also want to thank the staff of TSW Urology for their professionalism, support, and assistance with completion of this special edition.

* Evidence-Based Practice in Spina Bifida: Developing a Research Agenda, May 9-10, 2003, Washington DC

**This article should be cited as follows:**

Joseph, D.B. (2007). Current challenges in Spina Bifida care. *TheScientificWorldJOURNAL:* TSW Urology **7**, 1930–1931. DOI 10.1100/tsw.2007.263.
